# Plant-Plant-Microbe Mechanisms Involved in Soil-Borne Disease Suppression on a Maize and Pepper Intercropping System

**DOI:** 10.1371/journal.pone.0115052

**Published:** 2014-12-31

**Authors:** Min Yang, Yu Zhang, Lei Qi, Xinyue Mei, Jingjing Liao, Xupo Ding, Weiping Deng, Limin Fan, Xiahong He, Jorge M. Vivanco, Chengyun Li, Youyong Zhu, Shusheng Zhu

**Affiliations:** 1 Key Laboratory of Agro-Biodiversity and Pest Management of Education Ministry of China, Yunnan Agricultural University, Kunming, Yunnan, China; 2 Department of Horticulture and Landscape Architecture and Center for Rhizosphere Biology, Colorado State University, Fort Collins, Colorado 80523, United States of America; Estación Experimental del Zaidín (CSIC), Spain

## Abstract

**Background:**

Intercropping systems could increase crop diversity and avoid vulnerability to biotic stresses. Most studies have shown that intercropping can provide relief to crops against wind-dispersed pathogens. However, there was limited data on how the practice of intercropping help crops against soil-borne Phytophthora disease.

**Principal Findings:**

Compared to pepper monoculture, a large scale intercropping study of maize grown between pepper rows reduced disease levels of the soil-borne pepper Phytophthora blight. These reduced disease levels of Phytophthora in the intercropping system were correlated with the ability of maize plants to form a “root wall” that restricted the movement of *Phytophthora capsici* across rows. Experimentally, it was found that maize roots attracted the zoospores of *P. capsici* and then inhibited their growth. When maize plants were grown in close proximity to each other, the roots produced and secreted larger quantities of 2,4-dihydroxy-7-methoxy-2H-1,4-benzoxazin-3(4H)-one (DIMBOA) and 6-methoxy-2-benzoxazolinone (MBOA). Furthermore, MBOA, benzothiazole (BZO), and 2-(methylthio)-benzothiazole (MBZO) were identified in root exudates of maize and showed antimicrobial activity against *P. capsici*.

**Conclusions:**

Maize could form a “root wall” to restrict the spread of *P. capsici* across rows in maize and pepper intercropping systems. Antimicrobe compounds secreted by maize root were one of the factors that resulted in the inhibition of *P. capsici*. These results provide new insights into plant-plant-microbe mechanisms involved in intercropping systems.

## Introduction

Modern agricultural monocultures are highly productive but are usually vulnerable to disease, insects and climatic anomalies [1]. It has been suggested that increasing plant diversity in an ecosystem could augment its stability and avoid its vulnerability to biotic stresses [1]–[4]. Intercropping is the practice of growing two or more crops in the same field, and it is widely used in Asia, Latin America and Africa, providing as much as 15–20% of the world's food supply [5], [6]. Intercropping systems have been shown to increase yields despite the use of limited inputs such as fertilizers and fungicides [7]–[11]. Moreover, some studies have shown that intercropping can provide relief to crops against wind-dispersed pathogens, such as rice blast (caused by *Magnaporthe grisea*) [10] and cereal powdery mildew (caused by *Erysiphe graminis*) [12], [13]. The reduced disease severity found in these systems that have a more genetically diverse plant population may involve several mechanisms such as inoculum dilution, spore dispersal interference, micro-environmental modification, and induced resistance [12], [14]–[18]. However, there is limited data on how the practice of intercropping help crops against soil-borne pathogens.

*Phytophthora* is one kind of soil-borne pathogen that is difficult to control due to its wide host range and its ability to survive in the soil for long periods of time [19]. Although the use of resistant cultivars, different agricultural practices, fungicide applications, and other cultural methods have been recommended to control Phytophthora disease, none of these methodologies are sufficiently effective, practical, or economical [19]. The results of several field studies suggest that intercropping of maize with potato or pepper can suppress the incidence of Phytophthora disease and increase yield [8], [20]–[22]. However, the mechanisms by which maize is able to suppress soil-borne diseases of *Solanaceous* crops are unknown.

Here, we conducted a large scale intercropping field study of maize and pepper, combined with root interaction and root exudates analyses to determine the potential mechanisms involved in Phytophthora blight control under intercropping conditions. Maize roots and root exudates were found to play a crucial role in the control of Phytophthora disease on pepper plants.

## Materials and Methods

The field study was carried out in Wenshan and Yanshan counties of Wenshan Prefecture. These field studies were authorized by Plant Protection Station of Wenshan Prefecture, Yunnan, China. No specific permissions were required in these fields. The field study did not involve endangered or protected species.

### Effect of maize and pepper intercropping on yield and pepper Phytophthora blight

**Figure 1:**
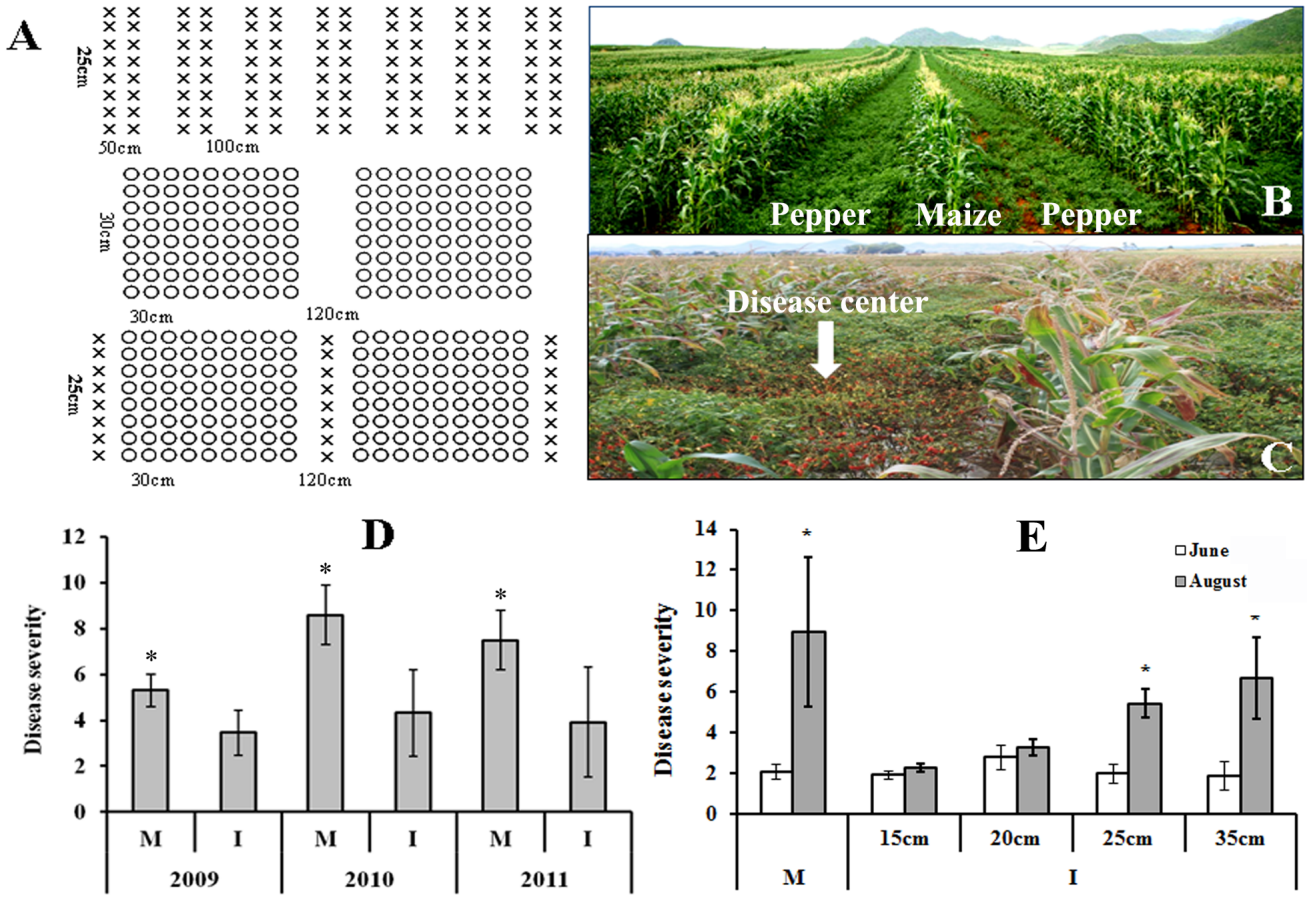
Maize and pepper intercropping in field and the effect on the development of pepper Phytophthora blight. (A) Maize and pepper intercropping and monoculture patterns. Each symbol represents a plant of a different crop species: maize (×), pepper (○). In maize monoculture system, the wide inter-row spacing was 100 cm and the narrow inter-row spacing was 50 cm. The intra-row spacing was 25 cm; In pepper monoculture system, the inter-row and intra-row spacing were 30 cm×30 cm in each strip. The space between strips was 120 cm. In maize and pepper intercropping system, the width of each strip was 3.4 m, and one row of maize intercropping with nine rows of pepper was planted in each strip. The inter-row and intra-row spacing for pepper plant was 30 cm×30 cm. The intra-row spacing of maize plant was 25 cm. The inter-row spacing between maize and pepper plants was 60 cm; (B) Maize and pepper intercropped in field; (C) Pepper Phytophthora blight in intercropping system. Arrow shows the disease center. Maize can restrict pepper Phytophthora blight across the maize line; (D) Disease severity (±SE) of pepper Phytophthora blight in monoculture and maize/pepper intercropping system from 2009 to 2011. Asterisks indicate statistically significant differences of monoculture compared to intercropping (Student's t test; *p*<0.05; n = 10); (E) Effect of maize with different intra-row spacing on the disease severity of pepper Phytophthora blight incidence (±SE) in intercropping system in 2012. Asterisks indicate statistically significant difference of severity surveyed in August and June (Student's t test; *p*<0.05; n = 5). M and I in figure D and E represent pepper monoculture and pepper intercropping with maize, respectively.

Three large scale field studies were conducted in China from 2009 through 2011 to examine the effect that intercropping of maize and pepper plants had on the disease severity of pepper Phytophthora blight. In 2009, the experiment was carried out on 135 hectares of maize and pepper intercropping farmland in the town of Jiangna in Yanshan County (N23.63°, E104.37°). In 2010, the experiment was carried out on 330 hectares of maize and pepper intercropping farmland in the town of Pinyuan in Yanshan County (N23.72°, E103.79°). In 2011, the experiment took place on 650 hectares of maize and pepper farmland in the town of Shupi in Qiubei County (N23.88°, E104.14°). In each location, three sites were selected at random for the experimental survey of yield and pepper Phytophthora blight. The experiment included three treatments at each site: an intercropping of maize and pepper, monocultures of pepper and monoculture of maize. Each treatment contained three, 200 m^2^ experimental plots arranged in the same field using a completely randomized block design. A schematic illustration of the planting arrangement is shown in Fig. 1A. The cultivars of maize and pepper used in these experiments are listed in S1 Table.

The actual yield per plot was determined as dry grain weight for maize and dry fruit weight for pepper. Land equivalent ratio (LER), which was used to evaluate the efficiency of intercropping with respect to monoculture, was determined according to the equation: LER  = Y_A_/M_A_+Y_B_/M_B_ where Y_A_ and Y_B_ are the individual crop yields per hectare in intercropping, and M_A_ and M_B_ are their yields per hectare as monoculture crops [23]. Crop values were based on market prices of 1616 US$ per ton for pepper and 291 US$ per ton for maize. The severity of pepper Phytophthora blight was assessed at five uniformly distributed survey sites per plot with a total of 300 plants assessed at each survey site. Disease severity was evaluated at mature stage of pepper using a 0–5 scale (0 =  no visible disease symptom; 1 =  leaves slightly wilted with brownish lesions beginning to appear on stems; 2 = 30–50% of entire plant diseased; 3 = 50–70% of entire plant diseased; 4 = 70–90% of entire plant diseased; 5 =  dead plant) (Sunwoo et al., 1996). Disease severity  =  [∑ (The number of diseased plants in this index × Disease index)/(Total number of plants investigated × The highest disease index)] ×100%. Two-tailed t-tests were used to determine if the Phytophthora blight severity differed significantly (p<0.05) between intercropping and monoculture field designs.

### Effect of maize spacing on pepper Phytophthora blight control

#### Field study

In order to determine whether maize plants could restrict the spread of *P. capsici*, the control efficacy of maize plant density on pepper Phtyophthora blight was tested in a field (23.86°N, 104.05°E) where Phytophthora blight was found to naturally occur in 2012. Using the maize Genyuan-135 and pepper WJ-3 cultivars, the experiment included five treatments: one treatment consisted of pepper plants grown in monoculture, while the other four treatments had maize planted at four different intra-row spacing distances (15, 20, 25, and 35 cm) in a maize/pepper intercropping system. A schematic illustration of these planting arrangements is shown in Fig. 1A. Each treatment was comprised of five, 50 m^2^ experimental plots. All plots were located in the same field and arranged using a randomized block design. The disease incidence of pepper Phytophthora blight was assessed on 300 plants in each of five uniformly distributed sites per plot. Disease severity of pepper Phytophthora disease was surveyed twice in June and September of 2012 according the method described above. Two-tailed t-tests were used to determine if the Phytophthora blight severity differed significantly (p<0.05) in August and June for each treatment.

#### Greenhouse study

**Figure 2:**
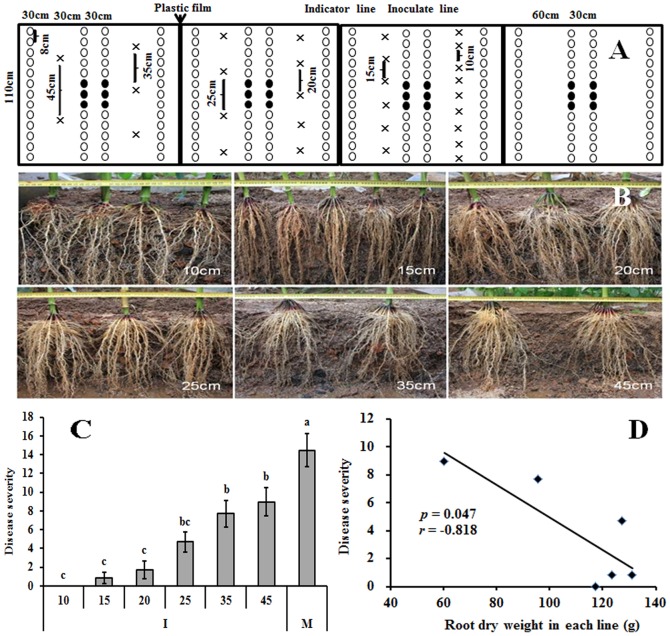
Effect of maize plant with different intra-row spacing on the root architecture and the spread of pepper Phytophthora blight in the greenhouse. (A) A schematic illustration of maize and pepper intercropping arrangements in the greenhouse. Maize planted with six different intra-row spacing distances (10, 15, 20, 25, 35 and 45 cm). Each plot contains two lines of maize with different intra-row spacing. Two lines of pepper were planted between the two lines of maize, and one line of pepper was sown outside of each maize line as an indicator line. Six peppers in the center of two pepper lines were inoculated with zoospores of *P. capsici*. The incidence of pepper Phytophthora blight in indicator lines was surveyed to show the ability of the zoospores to spread; (B) Changes in maize root architecture with different intra-row spacing distances at silking stage; (C) Effect of maize intra-row spacing on the spread of pepper Phytophthora blight in plastic houses. M and I represent pepper monoculture and pepper intercropping with maize, respectively. Means and standard errors are shown. Different letters indicate statistically significant differences analyzed using Turkey Post-Hoc ANOVA (p<0.05; n = 9); (D) The correlation analysis between disease incidence and maize root biomass in each line at silking stage.

The effect that different intra-row spacing maize plants can have on the spread of pepper Phytophthora blight was also studied in an intercropping system under greenhouse conditions that incorporated the additional parameter of maize root distribution. This experiment included seven treatments: one treatment consisted of pepper plants grown in monoculture, while the other six treatments had maize planted at six different intra row spacing distances (10, 15, 20, 25, 35 and 45 cm) in the intercropping system. A schematic illustration of these planting arrangements is shown in Fig. 2A. The area of each plot was 2.1 m×1.1 m, separated individually using plastic film. In the intercropping system, each plot had two rows of maize with different intra-row spacing. Two rows of peppers were planted between these two rows of maize, and one row of peppers was planted outside of each maize row as an indicator row. The inter-row spacing between maize and pepper plants was 30 cm. The inter- and intra-row spacing for pepper plants was 30 and 8 cm in both the monoculture and intercropping systems. Each treatment was replicated three times. All plots were located in the same greenhouse and arranged using a randomized block design. Seeds of maize (Genyuan-135) and pepper (WJ-3) were surface sterilized (6% H_2_O_2_, 10 min) and germinated on moist filter paper before being transplanted. Plots were weeded manually and irrigated with a spraying system to keep the soil moist. Once the maize plants reached the small bell-mouth stage, six centrally located pepper plants in the two rows between maize rows were inoculated with 10 mL of *P. cacpisi* zoospores (1×10^6^ zoospores/mL) per plant (Fig. 2A). Disease severity of pepper Phytophthora disease in the two peripheral indicator rows was surveyed according the above method to show the ability of zoospores to spread across maize roots.

The same treatments and replicates, with the exception of pepper monoculture, were set up as described above to observe the root distribution and biomass of maize at different intra row spaces. When maize plants reached the silking stage, a vertical cut was made in the soil profile 20 cm away from the rows of maize. The roots exposed with this cut were then carefully washed with water to remove soil particles. After taking photographs to record the root architecture (Fig. 2B) all roots per plant were sampled to measure biomass. Roots were dried at 85°C for 96 h to measure their dry weight. The effect of different intra-row spacing of maize on the spread of *P. capsici* was analyzed using Turkey Post-Hoc ANOVA (multiple comparisons) with PASW Statistics 18 (SPSS Inc.). The relationship between the root biomass of maize in each row and the incidence of pepper Phytophthora disease in the peripheral indicator rows was analyzed using Pearson correlation.

### Effect of maize spacing on the accumulation of DIMBOA and MBOA in roots and shoots

**Figure 3:**
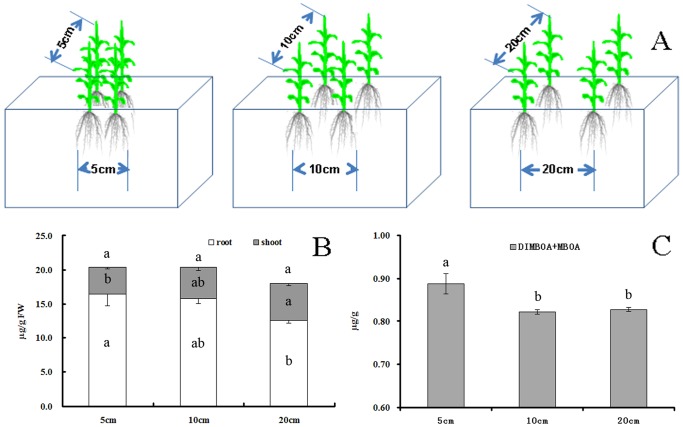
The accumulation and secretion of DIMBOA and MBOA in root under different maize plant distances. (A) A schematic illustration of maize grown at three distances 5 cm×5 cm, 10 cm×10 cm, and 20 cm×20 cm; (B) Accumulation of DIMBOA and MBOA in roots and shoots of maize grown at the three distances. Data represents mean values in µg g^−1^ fresh weight (FW) from three replicated samples; (C) The content of DIMBOA and MBOA in maize rhizosphere soil. Different letters designate significant differences analyzed using Turkey Post-Hoc ANOVA (p<0.05; n = 3).

The effects that plant distance might have on the accumulation of 2,4-dihydroxy-7-methoxy-2H-1,4-benzoxazin-3(4H)-one (DIMBOA) and 6-methoxy-2- benzoxazolinone (MBOA) in roots and shoots of maize was also tested. Maize was planted with different inter and intra row spacing distances inside plastic boxes in greenhouse. Each box (40 cm×30 cm×20 cm) was filled with 6 L of soil. Soils were prepared with a mix of 2 parts sand and one part pine soil (pine soil (pH 6.3; 1.4% organic matter; nutrient content: 2.6 mg/kg NH4-N, 3.6 mg/kg NO3-N, 3.0 mg/kg P, 136 mg/kg K, 1.1 mg/kg Zn, 16.7 mg/kg Fe, 2.5 mg/kg Mn, 1.3 mg/kg Cu). Four seeds were sown into each box at different inter and intra row spacing distances 5 cm×5 cm, 10 cm×10 cm, and 20 cm×20 cm, respectively (Fig. 3A). Each treatment had three replicates. All boxes were placed in the greenhouse and rotated every week. In the greenhouse, the temperature was controlled under 35°C. Plants were irrigated once initially with 1/2 strength Hoaglands solution (100 mL) [24], and then twice a week with water throughout the course of the experiment. When the maize plants reached the four leaf stage, they were removed from the boxes. Rhizosphere soil was collected and their roots were submerged in water to rinse away any sand residue. Two plants were randomly selected from each box for the analysis of DIMBOA and MBOA in roots and shoots according to the method outlined by Ahmad *et al.* [25] with a few modifications. Briefly, 5 g of plant roots or shoot samples were frozen in liquid nitrogen and powdered with a mortar and pestle. Pulverized tissue samples received 5 mL extraction buffer (methanol/acetic acid; v/v = 49/1), were sonicated (10 min) and then centrifuged (10,000 g, 10 min). The methanol fractions were dried under N_2_ gas and dissolved in 1 mL methanol. Rhizosphere soil collected from four plants was pooled together for one replication. The analysis of DIMBOA and MBOA in rhizosphere soil was performed using a published procedure [26] with a few modifications. Thirty grams of dry crushed soil was poured into a 50-mL extraction tube with 30 mL extraction buffer (methanol/acetic acid; v/v = 49/1). Samples were sonicated, centrifuged and dried using the same method as above. Reverse phase chromatography was used to separate 10 µL of each sample with a chromatographic system (Dionex Co., Sunnyvale, CA) equipped with a diode array detector. Solvent and column parameters were as follows: Solvent A = 0.05% glacial acetic acid (Mallinckrodt Chemicals, LC/MS grade) in water (Fisher Chemical, HPLC grade); Solvent B = 0.05% glacial acetic acid in methanol (OmniSolv, HPLC grade); column: C_18_, 4.6×250 mm 5 µm 120 Å (Dionex Co., Sunnyvale, CA); column temperature  = 35°C. The solvent gradient parameters were as follows: Flow rate: 0.7 mL/min; The gradient consisted of 0–1 min 3–20% solution B, 1–20 min, 20–100% solution B, and 20–35 min isocratic conditions of 100% solution B. The concentration (µg/g fresh weight) of DIMBOA and MBOA in maize tissues was calculated from standard curves, which showed linear relationship between peak area and concentration.

### Interaction assay between roots and zoospores

*P. capsici* (C-33) was grown on carrot agar medium (CAM) (200 g boiled carrot and 15 g agar in a total volume of 1 L distilled water), and zoospores were produced as described previously [27]. A modified capillary root model, as described by Fan [28], was used to monitor the interaction of roots and zoospores. Briefly, the capillary tube (1 mm external diameter) was bent into a U-shape, placed on a glass slide and overlaid with a coverslip to form a chamber with one open side. The primary roots were obtained from maize (cv. Genyuan-135), maize DIMBOA mutant *Bx1* (supplied by the Maize Genetics Cooperation Stock Center of USDA/ARS & Crop Sciences/UIUC), and pepper seeds (cv. WJ-3) that had been surface-sterilized for 5 min in sodium hypochlorite solution (1% w/v available chlorine), thoroughly washed in sterile water and allowed to germinate on moist filter paper in petri dishes in darkness at 24°C. When the primary roots reached about 2 cm long, the primary roots were excised with a sterile razor blade. The tip of each root was then inserted into the open end of the chamber, and zoospore suspensions (1×10^4^ zoospores/mL) were introduced into the chamber. The slides were incubated in a humid petri dish at room temperature. The behavior of zoospores on root tip, elongation zone, root hair zone, and at 5 mm away from the root tip were recorded every five minutes for a period of 30 minutes using a video camera attached to a compound microscope (Leica DM2000, Germany). A capillary tube was inserted into a chamber with the same zoospore suspension as a control. The number of zoospores, cystospores, germinated and ruptured cystospores on the different root zones were all counted on photographs. The percentage of zoospores encysted into cystospores, and germinated or ruptured cystospores was calculated. Chemotactic ratio (CR) was determined following the formula described by Halsall (1976) where CR  =  scores of zoospores and cystospores on test root/score of zoospores and cystospores on control. Positive CR values indicate positive chemotaxis [29]. The experiment was repeated three times, and five roots were tested per run.

### Root exudates collection and identification

#### Root exudates collection

Root exudates of maize (cv. Haihe-1 and Genyuan-135) were collected by a trapping system as previously described with a few modifications [30]. Briefly, maize seeds were sterilized (6% H_2_O_2_, 10 min) and sowed into washed silica sand in glass pots (2 L sands per pot). One seed was sowed into each pot in the greenhouse and irrigated with 0.1 strength Hoagland solution at a rate of 10 mL/day. Additional distilled H_2_O was supplied as needed. When the maize plants reached the four leaf stage, each pot was washed with 2 L of distilled H_2_O. A column filled with Amberlite XAD-4 reins (Sigma-Aldrich, Beijing) and the circulating attachment was then connected to the trapping systems. The solution was circulated at a rate of 1 L/h by airlift. The root exudates from each plant were collected in separate columns, with 15 plants and columns in total. The column was detached after 7 days, washed with 10-bed volumes of distilled H_2_O, and then eluted with 200 mL methanol (OmniSolv, HPLC grade) followed by 100 mL dichloromethane (Fisher Chemical, HPLC grade). Elutes from 15 columns were pooled, filtered and concentrated under reduced pressure. The concentrate was dissolved into 1 mL of methanol for further analysis. This trapping system was used without maize plants as control.

#### Gas chromatography-mass spectrometry (GC-MS) analyses of root exudates

The GC/MS fingerprints of root exudates were obtained using an Agilent 7890–5975 (USA). Root exudates were dried under nitrogen gas followed by methoximation and trimethylsilylation derivatization as described by Xu *et al.* [31]. Root exudates were separated in an HP-5 MS capillary column (19091S-433, 30 m×0.25 mm×0.25 µm, Agilent). The injector operating conditions were as follows: injection volume was 1 µL in the splitless mode; injector temperature was 260°C. The initial column temperature was 40°C (held 2 min) and programmed to increase at a rate of 5°C/min to 250°C and then held for 10 min. The transfer line temperature was 280°C. Helium (purity of 99.999%) was used as a carrier gas with a flow rate of 1 mL/min. Electronic impact (EI) ionization mode mass spectra were obtained at 70 eV and monitored on the full-scan range (*m/z* 50–550). Mass fragments of the components were compared to the mass fragmentation data contained in the NIST/US Environmental Protection Agency (EPA)/National Institutes of Health (NIH) Mass Spectral library (NIST 05). Components with more than 80% confidence rate were regarded as undoubtedly existing in the root exudates. The components that appeared in blank tests were not recorded in the final result.

#### High performance liquid chromatography-mass spectrometry (HPLC–MS) analysis of root exudates

Three compounds, BZO, MBZO and MBOA, were further selected to determine their presence in maize root exudates by HPLC-MS. BZO and MBZO were purchased from J & K Beijing Scientific GmbH. MBOA was purchased from Sigma-Aldrich Shanghai Trading Co. Ltd. Maize root exudates were analyzed using an HP 1100 HPLC system fitted with a diode array detector and directly connected to a Bruker Esquire HCT Esquire 3000 electrospray ionization (ESI) ion trap mass spectrometer (Bruker Daltonik GmbH, Shanghai, China) working in the positive ion mode. The HPLC separations were performed on an Agilent Zorbax SB-C18 column (4.6×150 mm, 5 µm). Solvent was as follows: Solvent A = 0.05% glacial acetic acid (Mallinckrodt Chemicals, LC/MS grade) in water (Fisher Chemical, HPLC grade); Solvent B = 0.05% glacial acetic acid in methanol (OmniSolv, HPLC grade). A multistep gradient was used for all separations with an initial injection volume of 2 µL and a flow rate of 0.8 mL/min. The multistep gradient was as follows: 0–30 min 30–60% (v/v) solution B, and 30–40 min isocratic conditions of 60% solution B. The column temperature was maintained at 35°C. Chromatograms were recorded at 230 nm and retention times of DIMBOA, MBOA and MBZO were established from standards. The ESI-MS conditions were as follows: nitrogen (N_2_) was used as nebulizer gas (25 psi) and as drying gas (10 L/min N_2_ at 330 °C). The capillary voltage was set at 4000 V, the capillary exit at 106 V, and the skimmer at 40 V; spectra were recorded at normal resolution (0.6 u full width at half-peak height), under ion charge control (ICC) conditions (100 000) in the mass range from m/z 25 to 1000 and 36.8 V trap drive value.

#### Inhibition of *P. capsici* by root exudates and pure compounds

The inhibitory activity of root exudates and target compounds (MBOA, BZO, and MBZO) against the release of zoospores from sporangia, zoospores motility, cystospores germination and rupture was measured according to a published procedure [32] with a few modifications. Briefly, an aliquot of 40 µL of root exudates at concentrations of 0, 0.06, 0.10, 0.5, 1.0, and 5.0 mg/mL or a target compound solution (MBOA, BZO, or MBZO) at concentrations of 0, 0.02, 0.1, 0.2, 0.6, and 1.0 mg/mL was added to depression glass slides. Then, 40 µL of sporangia suspension (1×10^4^ sporangia/mL), zoospores suspension (1×10^5^ zoospores/mL), or cystospores suspension (1×10^5^ cystospores/mL) was added immediately to the glass slides containing each respective solution. Slides were placed in Petri dishes containing moist filter paper and incubated in darkness at 24°C. The percentage of empty sporangia was visualized under a microscope after sporangia incubation for 2 h. The percentage of zoospores encysted into cystospores was recorded under the microscope after zoospore incubation for 20 min. The percentage of germinated and ruptured cystospores was counted under a microscope after cystospore incubation for 4 h. The experiment was conducted three times, each time in triplicate.

The inhibitory activity of root exudates and target compounds on colony growth of *P. capsici* was determined according to a previously published method [33]. Briefly, a fresh plug (5 mm in diameter) was taken from the growing edge of a carrot agar medium (CAM) (200 g boiled carrot and 15 g agar in a total volume of 1 liter of distilled water) culture and transferred onto CAM supplemented with root exudates (0, 0.06, 0.10, 0.5, 1.0, and 5.0 mg/mL) or target compound (MBOA, BZO, or MBZO) (0, 0.02, 0.1, 0.2, 0.6, and 1.0 mg/mL), respectively. In all cases, the final amount of solvent never exceeded 1% (vol/vol) in treated and control samples. Colony growth was assessed by measuring the increase in colony diameter after incubation in darkness at 25°C for 4 days. The inhibition of root exudates or target compounds against colony growth was calculated [33].

## Results

### Maize and pepper intercropping increase yield and decrease pepper Phytophthora blight

The crop value from maize and pepper intercropping were higher compared to those from maize or pepper monocultures (S1 Table), resulting in land equivalent ratios (LER) of 1.81, 1.45 and 1.42 in 2009, 2010, and 2011 respectively. The severity of Phytophthora blight on pepper plants in the intercropping system was significantly reduced by 33.5±6.6%, 49.1±7.0% and 46.0±10.6% in 2009, 2010, and 2011 (p<0.05), respectively when compared to the peppers grown in monoculture (Fig. 1D and S1 Table). Additionally, it was observed that Phytophthora blight did not spread across maize rows to infect the other rows of peppers (Fig. 1C).

### Maize roots block the spread of *P. capsici* in the soil

Further field studies showed that the disease severity surveyed in August in pepper monoculture significantly increased compared with the initial disease severity surveyed in June (Fig. 1E). An intercropping system with maize having an intra-row spacing of 15 or 20 cm did not show significantly increased disease severity; however, maize having an intra-row spacing of 25 and 35 cm showed higher disease severity compared with the initial disease severities surveyed in June (Fig. 1E).

Greenhouse studies corroborated that intra-row spacing of maize in the intercropping system affected the spread of pepper Phytophthora blight (Fig. 2C). Maize with an intra-row spacing of 35 and 45 cm showed a significantly lower ability to restrict the spread of Phytophthora blight to peppers than maize plants that were sown at distances of 10, 15, and 20 cm (*p*<0.05). In particular, the disease was totally blocked when maize plants were separated by only 10 cm.

Further analysis indicated that the ability of maize to restrict the spread of pepper Phytophthora blight was correlated with its root distribution. Maize roots could contact each other to different degrees at the silking stage depending on their intra row spacing (Fig. 2B). The correlation analysis indicated that there was a significantly negative correlation between disease severity and maize root biomass in each row at the silking stage (*r* = −0.818, *p* = 0.047) (Fig. 2D).

### Maize root interactions induce the accumulation of DIMBOA and MBOA in the roots and rhizosphere soil

Previous studies have reported the antifungal compounds DIMBOA and its degradation product MBOA to be prevalent in maize [34]. In our studies, the accumulation of DIMBOA and MBOA in roots and rhizosphere soil was affected by plant distance. The concentration of DIMBOA and MBOA in roots and rhizosphere soil of plants separated by 5 cm was significantly higher than in plants separated by 10 cm and 20 cm (Fig. 3B–C). These data indicated that when maize roots are in close contact, the accumulation and secretion of DIMBOA and MBOA in roots tends to increase.

### Maize root interfere with the behavior and development of zoospores

**Figure 4:**
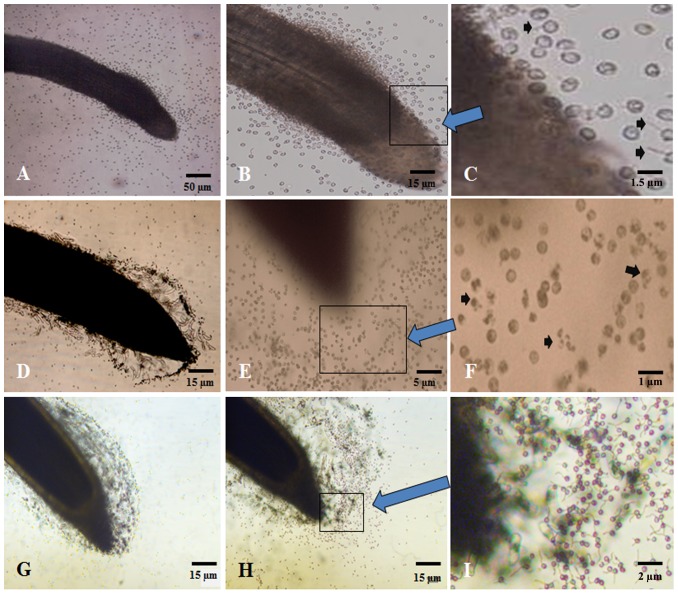
Interaction of pepper and maize roots with *Phytophthora capsici* zoospores. A∼C shows the interaction of pepper root and zoospores. Zoospores were attracted to the root elongation zone of pepper, quickly stopped and encysted into cystospores on the root surface or near the root. And then cystospores began to germinate. Arrow in C shows the germinated spores. D∼F shows the interaction of maize root and zoospores. Zoospores were attracted to the root tip of maize, quickly stopped and encysted into cystospores. Few cystospores germinated and some cystospores on the root tip of maize ruptured. Arrow in F shows the ruptured cystospores. G∼I shows the interaction of zoospores and the root of maize BX1 mutant, which can not produce DIMBOA and MBOA. Zoospores were also attracted to the root tip zone, quickly stopped and encysted into cystospores. However, most of cystospores did not rupture but germinated after 30 min incubation. Arrow in I shows the germinated cystospores.

The zoospores swam towards the roots of both pepper and maize and then attached to their surfaces (Fig. 4A–D). However, the attraction of zoospores to pepper roots was stronger than that to maize roots (S1A Fig.). In addition, zoospores preferred to swim to the root elongation zone of pepper. After attraction to the roots of maize and pepper, the zoospores quickly stopped and encysted into cystospores on the root surface or near the root (Fig. 4B and E). Zoospores completely encysted into cystospores after 30 min on the root tip of pepper and on the root elongation zone of maize (S1B Fig.). However, zoospores which were 5 mm away from the root tip of maize stopped and encysted more slowly, having less than 40% of zoospores encysted into cystospores even after 30 min (S1B Fig.). After cyst formation, cystospores on or near pepper root surfaces began to germinate (Fig. 4C) at a rate of 70% and 40% after 30 min on the root tip and elongation zone of pepper, respectively (S1C Fig.). However, few cystospores germinated on or near the maize root. In fact, 55.86% of cystospores on the root tip of maize ruptured after 30min (Figs. 4F and S1D Fig.). When zoospores interacted with the root of *Bx1* mutant, which cannot produce DIMBOA, zoospores were also attracted to the root tip zone, then quickly stopped and encysted into cystospores (Fig. 4 G). However, most of cystospores on the root tip of *Bx1* mutant germinated after 30 min incubation, and only 6.28% of cystospores ruptured, which was significantly lower than the wild type strain (*p*<0.05) (Fig. 4 H and I).

### Maize root exudates show inhibitory activity against different growth stages of *P. capsici*

**Figure 5:**
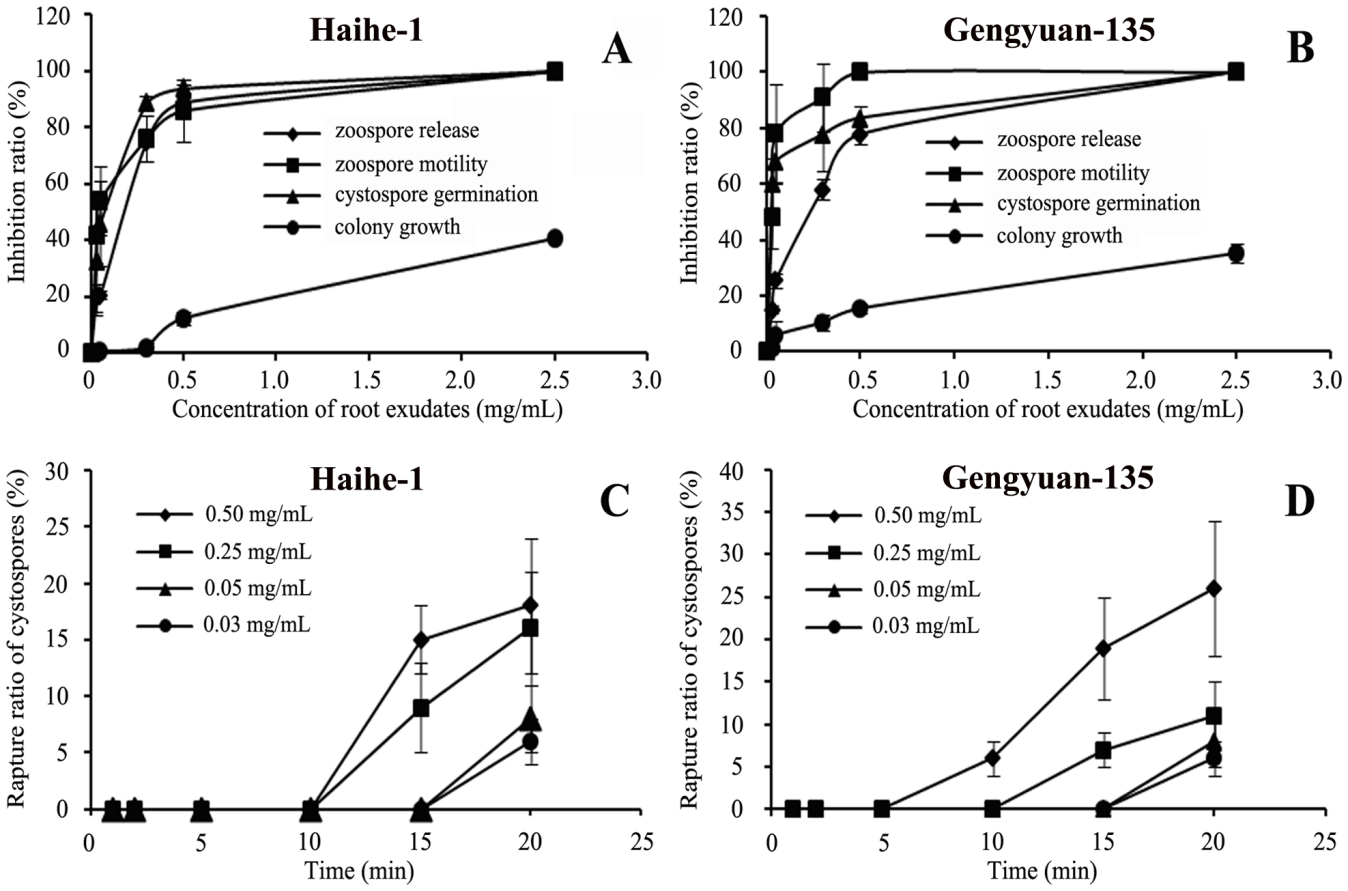
The inhibitory activity of maize root exudates on different life stages of *Phytophthora capsici*. A and B show the inhibitory activity of root exudates collected from the variety Haihe-1 and Genyuan-135 against the zoospore release, motility, cystospore germination, and colony growth of *P. capsici*. C and D show the effect of root exudates of Haihe-1 and Genyuan-135 on cystspore rupture, respectively. Error bars indicate SE (n = 3) of three replicates.

The root exudates of maize showed inhibitory activity against the release of zoospores from sporangia, zoospore motility, cystospore germination, and hyphal growth of *P. capsici* (Fig. 5A and B). Upon the treatment of sporangia with 0.5 mg/mL root exudates from maize Haihe-1 and Genyuan-135 varieties, the release of zoospores from the sporangia was inhibited by 88.9% and 77.7%, respectively. After exposure for 5 min to 2.5 mg/mL of Haihe-1 root exudates and 0.50 mg/mL of Genyuan-135 root exudates, zoospore motility was completely inhibited (Fig. 5A and B). Root exudates also showed dose-respondent inhibitory effects on cystospore germination and hyphal growth (Fig. 5A and B). The zoospore release, motility and spore germination were more sensitive to root exudates in contrast to its hyphal growth. The exposure to maize root exudates also caused a number of the cystospores to rupture. The percentage of ruptured cystospores reached to 18% and 26% after a 20 min exposure to 0.50 mg/mL of root exudates from Haihe-1 and Gengyuan-135, respectively (Fig. 5C and D).

### Compound identification in root exudates

GC/MS analysis identified a total of 10 and 17 compounds, respectively from the root exudates of the maize varieties Haihe-1 and Genyuan-135 (S2 Table). Among these compounds, MBOA, BZO, MBZO, 2-ethoxyethyl acetate, 3-methyl-2(3H) benzothiazolethione, 2(3H)-benzothiazolone, 1,4-Dimetyl-7-(1-methylethyl)-azulene, and dibutyl phthalate were present in the exudates of both varieties. As mentioned, previous studies have reported the antifungal activity of the compounds BZO, MBZO, and MBOA [34], [35]; therefore, these three compounds were further analyzed in the roots exudates of Haihe-1 and Genyuan-135 with HPLC-MS and successfully identified in both varieties. Three peaks at retention time (*t*_r_) 11.302, 17.7872 and 33.431 min in root exudates of Haihe-1 were identified in accordance with the purchased reference standards for MBOA, BZO and MBZO, respectively (S2A Fig.). ESI-MS data were collected at the corresponding retention times. Characteristic ESI-MS peaks for MBOA (*t*_r_ = 11.541–11.614 min, [M+H]^+^ = *m/z* 166, S2B Fig.), BZO (*t*_r_ = 17.997–18.195 min, [M+H]^+^ = *m/z* 136, S2C Fig.), and MBZO (*t*_r_ = 33.534 min, [M+H]^+^ = *m/z* 182, S2D Fig.) were used as the basis for their positive identification. These three compounds were also identified in root exudates of Genyuan-135 with similar ESI-MS data (S3 Fig.).

### The inhibitory activity of compounds in root exudates against *P. capsici*

**Figure 6:**
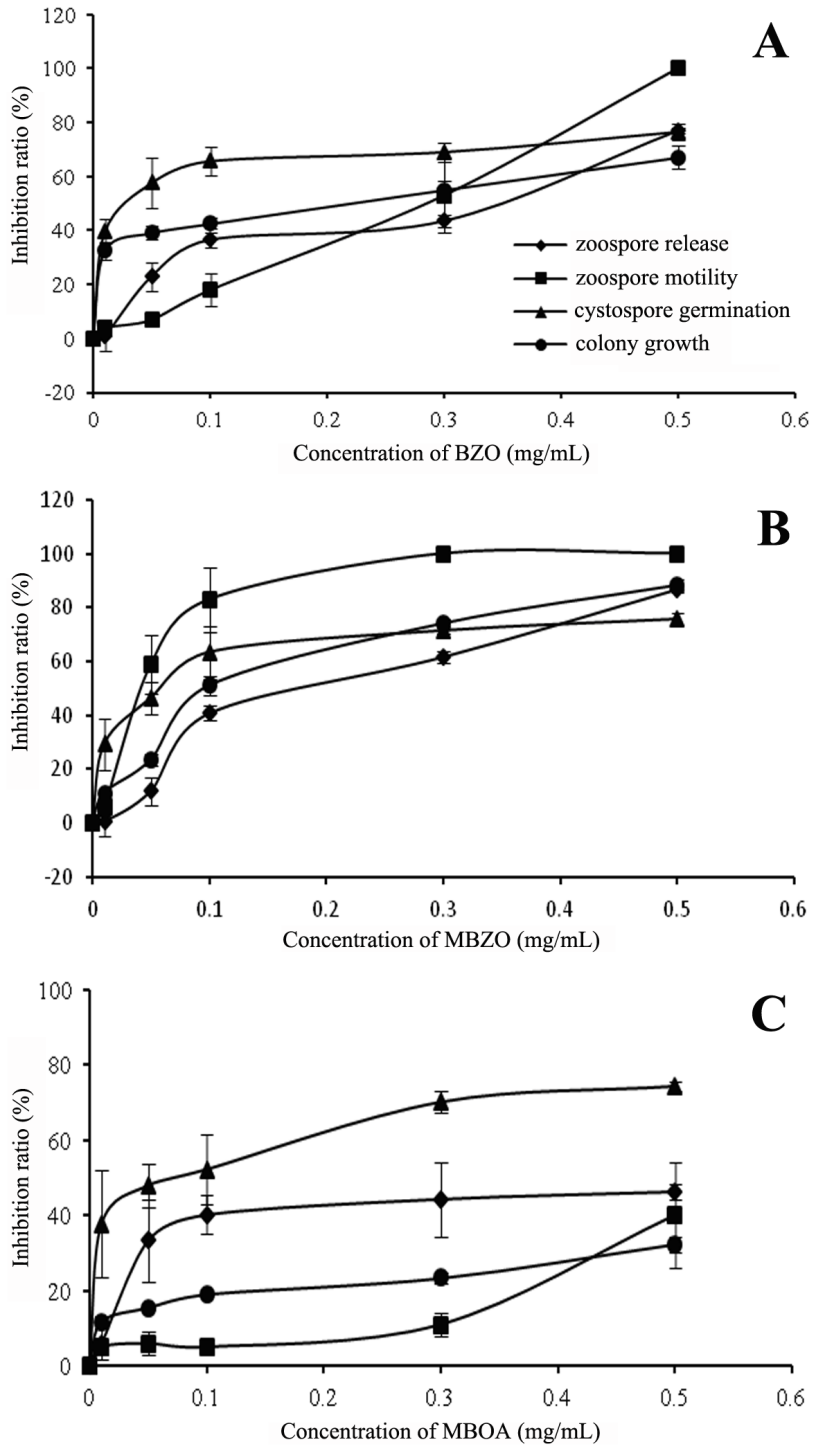
The inhibitory activity of compounds BZO (A), MBZO (B) and MBOA (C) against the release of zoospores from sporangia, zoospore motility, cystospore germination and colony growth of *Phytophthora capsici*. Error bars indicate SE (n = 3) of three replicates.

MBOA, BZO and MBZO showed dose-respondent inhibitory effects on zoospore release from sporangia, zoospore motility, cystospore germination, and hyphal growth of *P. capsici* (Fig. 6). Specifically, BZO indicated the highest inhibitory activity against cystospore germination and hyphal growth (Fig. 6A). MBZO demonstrated the strongest activity against zoospore motility which was completely inhibited after 5 min at a concentration of 0.3 mg/mL (Fig. 6B). This compound also demonstrated high activity against zoospore release from sporangia, cystospore germination and hyphal growth. MBOA showed slight activity against zoospore motility and hyphal growth, but showed high activity against cystspore germination (Fig. 6C).

## Discussion

This large-scale field experiment confirmed previous reports that maize and pepper intercropping increases total yields and reduces disease [20]. In conventional practice, pepper monoculture typically leads to outbreaks of pepper Phytophthora blight. Intercropping of maize with pepper, however, restricted the spread of pepper Phytophthora blight and resulted in higher total yields. Some studies indicated that intercropping can help crops against some soil borne pathogens, such as fungi [36], [37], [38], bacteria [39], and nematode [40]. To the best of our knowledge, this is the first report on intercropping for soil-borne Phytophthora disease control.

Here, we found that the effectiveness of maize and pepper intercropping in controlling Phytophthora correlated to the intra-row spacing between maize plants. When maize grows at low intra-row spacing, the roots can tightly touch each other to form a “root wall” that restricts the spread of Phytophthora blight to other rows of pepper plants. Our field and greenhouse studies demonstrated that the closer the maize plants are located to each other in each row in the intercropping systems, the higher their effectiveness to restrict the spread of pepper Phytophthora blight. This may not only be due to the physical barrier (root wall), but also due to the secretion of defense compounds by roots. The accumulation of DIMBOA and MBOA in roots and rhizosphere soil was increased when maize plants were grown in close proximity to each other. The compounds DIMBOA and MBOA are major secondary metabolites involved in maize defense against herbivorous insects and pathogens [34]. Previous studies indicated that the accumulation of DIMBOA in maize could be induced by pathogens [41] and insects [25], [41], or by the treatment with jasmonic acid [42]. To the best of our knowledge, there are no reports in the literature showing that the production of DIMBOA is induced by root-root proximity or interactions. Further analysis indicated that the induced accumulation of DIMBOA and MBOA in roots was a consequence of translocation from the shoots, which may benefit for the competition of maize under high density (Fig. 3B). A previous study demonstrated that when the primary leaf of wheat was infested by aphids, some benzoxazinoid hydroxamic acids were translocated from the roots and stems to the primary leaf [43]. Some literature highlights that when some plants are grown with conspecific neighbors they produce increased levels of defense-related secondary metabolites or proteins as compared to when grown alone or with heterospecific neighbors [44], [45]. This reaction might be due to root interactions and the actual interaction of roots might be based on physical touch [46] or by signals released by roots, such as proteins or metabolites present in the root exudates [44], [45], [47].

Maize roots can attract zoospores of *P. capsici* and secrete antimicrobial substances to kill them. *P. capsici* is a typical soil-borne pathogen that infects plants through the production of zoospores, which involves a pre-penetration process of zoospore taxis, encystment, cystospore germination and orientation of the germ-tube [19], [48]. Some studies indicated that the processes that occur before penetration of the cystospore inside the root are not host-specific [49]. For instance, chemotaxis and electrotaxis are involved in the attraction of zoospores to host and non-host roots [50]–[52]. Indeed, our study demonstrated that both pepper and maize roots could attract zoospores to their surface where they encysted into cystospores, but interestingly, those attracted by maize roots could not germinate or infect. This indicates that maize roots can attract zoospores and at the same time secrete antimicrobial substances against them. Here, it was found that root exudates of two maize varieties inhibited the release of zoospore from sporangia and zoospores' motility, even causing the rupture of many cystospores. Specifically, MBOA, BZO and MBZO were identified in the root exudates of maize. DIMBOA is prevalent in maize tissues and can also be secreted into rhizosphere soil by roots [53], [54]. Once secreted, DIMBOA degrades relatively quickly into MBOA in aqueous environments [55]. MBOA is considerably more stable in sterile soil [56], and has activity against fungi, insects and nematodes [34], [57]. It is evident in our study that MBOA was identified in maize root exudates and rhizosphere soil and showed inhibitory activity against the release of zoospores from sporangia, zoospore motility, cystospore germination and hyphal growth (Fig. 6). BZO and MBZO were also identified in maize root exudates. These two compounds have previously been identified in the root exudates of onion [58], *Potamogeton maachianus* [59], apple [60], rice [61], and *Zapoteca formosa* [62]. BZO was also detected in volatiles emitted from mechanically damaged poplar cuttings [63] and coyote tobacco (*Nicotiana attenuate*) leaves [64], as well as from some soil bacteria (such as *Bacillus, Stenotrophomonas*, *Arthrobacter*, *Ensifer*, *Sporosarcina*) [65]. Previous studies indicated that compounds containing the benzothiazole nucleus possess biological activities such as antimicrobial and anticancer agents [35]. The inhibitory activity of BZO and MBZO was also found to be effective on *P. capsici*. BZO showed high inhibitory activity against cystospore germination and hyphal growth, and MBZO showed high activity against the motility of zoospores. However, no compounds were identified that are specifically involved in the rupture of cystospores. Thus, there are some other compounds in maize root exudates that need to be further identified and characterized.

In conclusion, maize and pepper intercropping can effectively control the spread of Phytophthora blight in the field. The reduced disease levels of Phytophthora in the intercropping system were correlated with the ability of maize to form a “root wall” to restrict the spread of pepper Phytophthora blight across rows. Antimicrobial compounds secreted by maize appear to be strongly associated with inhibition of *P. capsici*. However, other alternative explanations such as physical barriers, the potential induction of beneficial Phytophthora-inhibiting microorganisms by the maize root, or the induced defense of maize by Phytophthora need further exploration. These results provide new insights into plant-plant-microbe mechanisms involved in intercropping systems.

## Supporting Information

S1 Fig **Interaction of zoospores of** ***Phytophthora capsici*** **with pepper and maize root.** (A) Taxis response of zoospores to pepper and maize root; (B) Encystment rates of zoospores in different zones of maize and pepper root; (C) The germination rate of cycstospore in different zones of maize and pepper root; (D) The rupture rate of cystospores in different zones of mazie and pepper root. Error bars indicate SE (n = 3) of three replicates.
(TIF)

S2 Fig **Separation and characterization of MBOA, BZO and MBZO from maize root exudates of Haihe-1 by high performance liquid chromatography (HPLC)-mass spectrometry (MS) analysis.** (A) HPLC profiles of root exudates showing three peaks at retention times (tr) 11.302, 17.7872 and 33.431 min in the root exudates of Haihe-1 were in accordance with the purchased reference standards MBOA, BZO and MBZO, respectively. (B) ESI-MS data was collected at 11.541–11.614 min. The characteristic peak for MBOA ([M+H]^+^ = m/z 166) was evident. (C) ESI-MS data was collected at 17.997–18.195 min. The characteristic peak for BZO ([M+H]^+^ = m/z 136) was evident. (D) ESI-MS data was collected at 33.534 min. The characteristic peak for MBZO ([M+H]^+^ = m/z 182) was evident.
(TIF)

S3 Fig **Separation and characterization of MBOA, BZO and MBZO from maize root exudates of Genyuan-135 by high performance liquid chromatography (HPLC)-mass spectrometry (MS) analysis.** (A) HPLC profiles of root exudates showing three peaks at retention times (tr) 11.2831, 17.6962 and 33.4174 min in root exudates of Genyuan-135 were in accordance with the purchased reference standards MBOA, BZO and MBZO, respectively. (B) ESI-MS data was collected at 11.382–11.522 min. The characteristic peak for MBOA ([M+H]^+^ = m/z 166) was evident. (C) ESI-MS data was collected at 17.911 min. The characteristic peak for BZO ([M+H]^+^ = m/z 136) was evident. (D) ESI-MS data was collected at 33.571 min. The characteristic peak for MBZO ([M+H]^+^ = m/z 182) was evident.
(TIF)

S1 Table **Disease control, yield and monetary value in monocultural and intercropping system.**
(DOCX)

S2 Table **Compounds identified by GC/MS analysis in root exudates of maize variety Haihe-1 and Genyuan-135.**
(DOCX)
