# Supplementary figures and images for: Plant-Plant-Microbe Mechanisms Involved in Soil-Borne Disease Suppression on a Maize and Pepper Intercropping System
Source: PLoS One. 2014 Dec 31;9(12):e115052. doi: 10.1371/journal.pone.0115052 (PMC4281244; doi:10.1371/journal.pone.0115052)

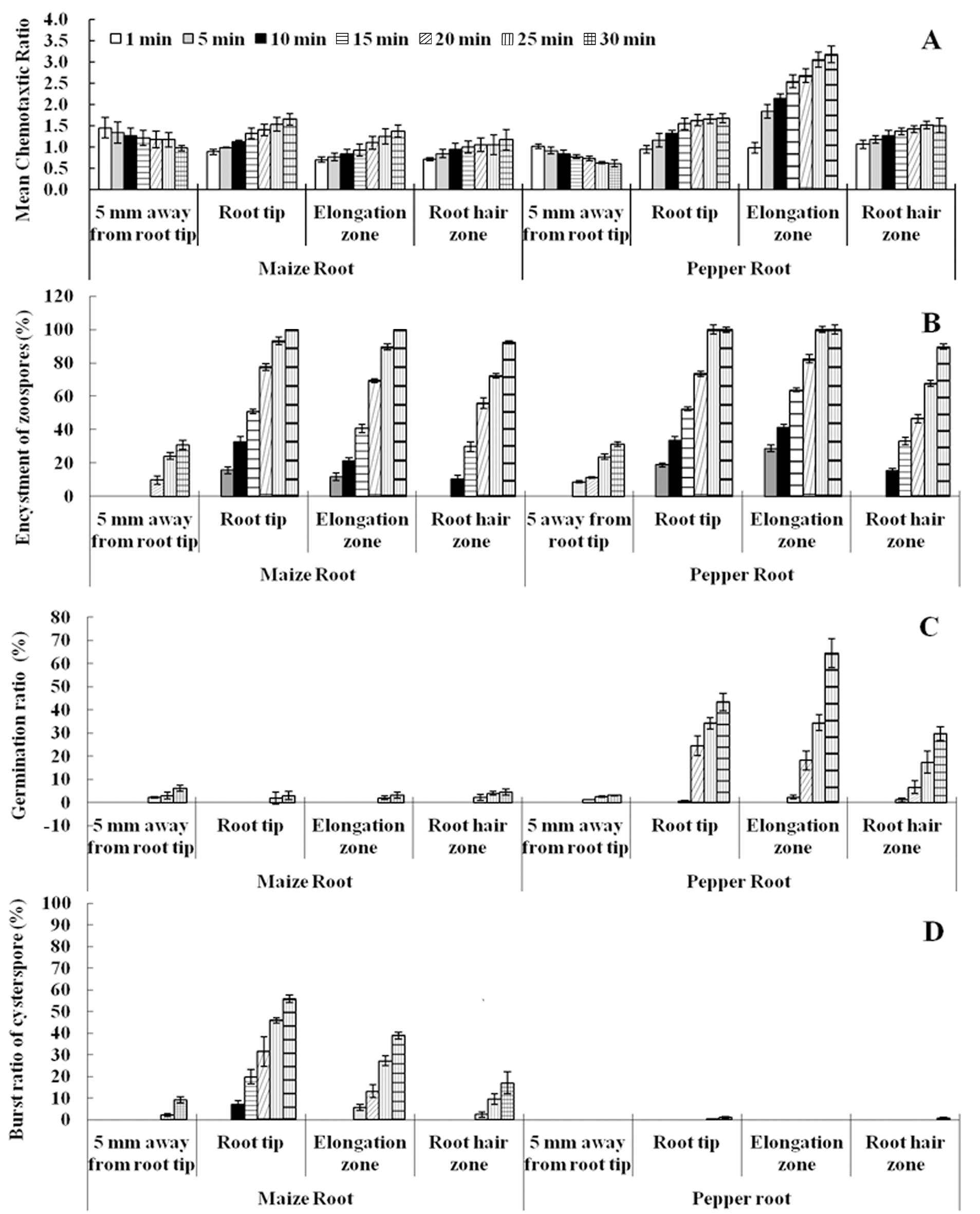

Supplement: S1 Fig — Interaction of zoospores of Phytophthora capsici with pepper and maize root. (A) Taxis response of zoospores to pepper and maize root; (B) Encystment rates of zoospores in different zones of maize and pepper root; (C) The germination rate of cycstospore in different zones of maize and pepper root; (D) The rupture rate of cystospores in different zones of mazie and pepper root. Error bars indicate SE (n = 3) of three replicates. (TIF) [file pone.0115052.s001.tif]

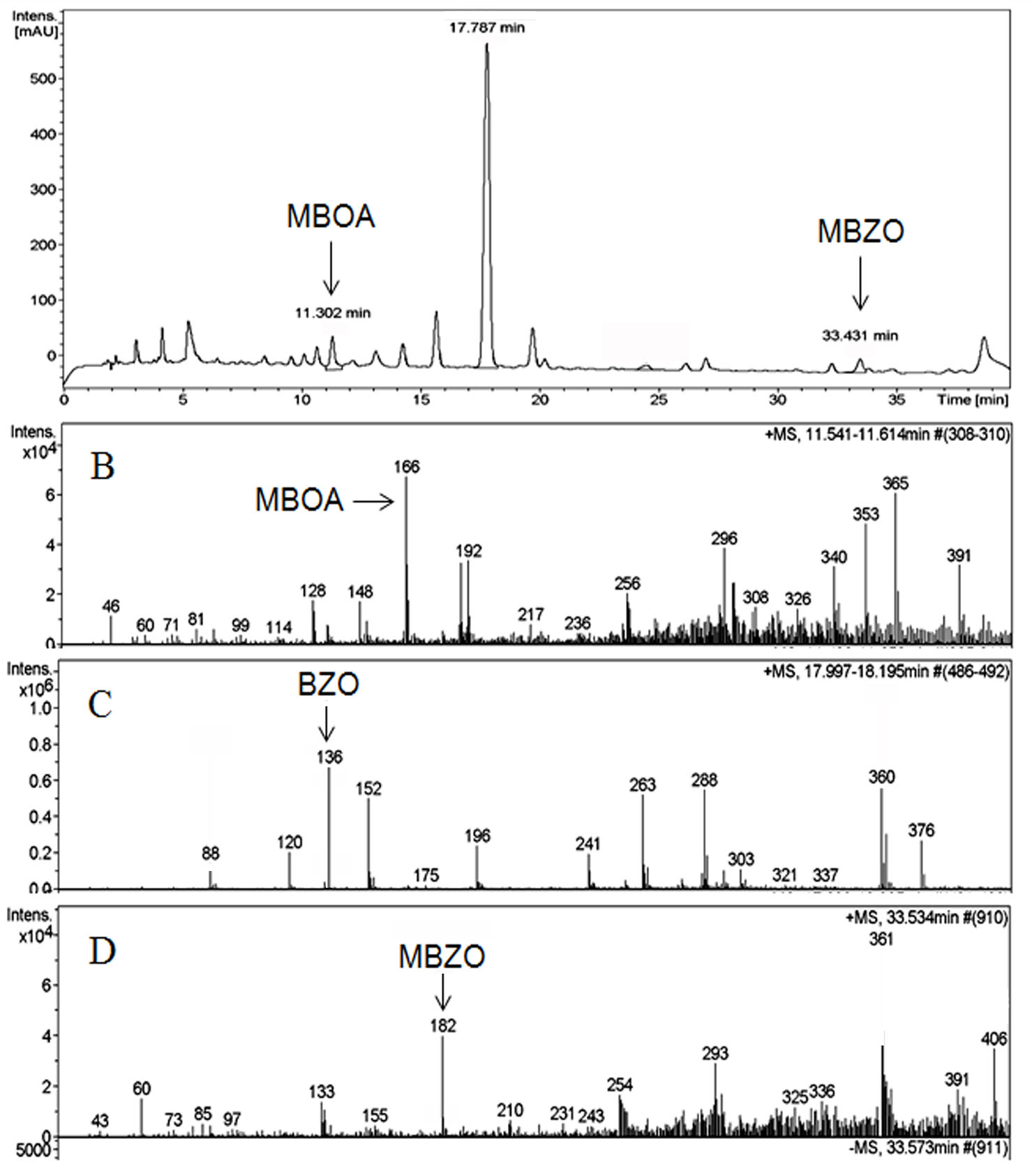

Supplement: S2 Fig — Separation and characterization of MBOA, BZO and MBZO from maize root exudates of Haihe-1 by high performance liquid chromatography (HPLC)-mass spectrometry (MS) analysis. (A) HPLC profiles of root exudates showing three peaks at retention times (tr) 11.302, 17.7872 and 33.431 min in the root exudates of Haihe-1 were in accordance with the purchased reference standards MBOA, BZO and MBZO, respectively. (B) ESI-MS data was collected at 11.541–11.614 min. The characteristic peak for MBOA ([M+H]+ = m/z 166) was evident. (C) ESI-MS data was collected at 17.997–18.195 min. The characteristic peak for BZO ([M+H]+ = m/z 136) was evident. (D) ESI-MS data was collected at 33.534 min. The characteristic peak for MBZO ([M+H]+ = m/z 182) was evident. (TIF) [file pone.0115052.s002.tif]

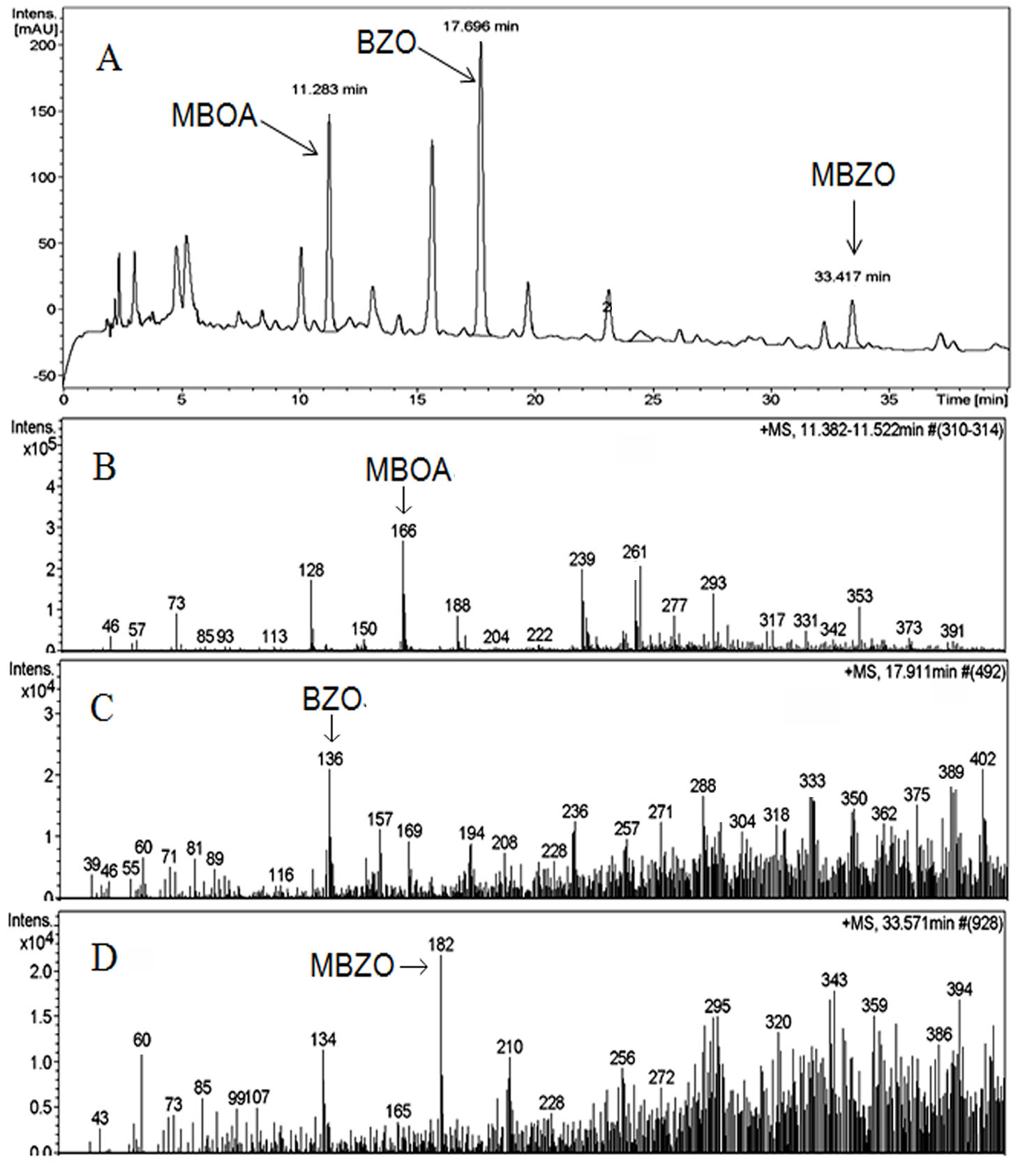

Supplement: S3 Fig — Separation and characterization of MBOA, BZO and MBZO from maize root exudates of Genyuan-135 by high performance liquid chromatography (HPLC)-mass spectrometry (MS) analysis. (A) HPLC profiles of root exudates showing three peaks at retention times (tr) 11.2831, 17.6962 and 33.4174 min in root exudates of Genyuan-135 were in accordance with the purchased reference standards MBOA, BZO and MBZO, respectively. (B) ESI-MS data was collected at 11.382–11.522 min. The characteristic peak for MBOA ([M+H]+ = m/z 166) was evident. (C) ESI-MS data was collected at 17.911 min. The characteristic peak for BZO ([M+H]+ = m/z 136) was evident. (D) ESI-MS data was collected at 33.571 min. The characteristic peak for MBZO ([M+H]+ = m/z 182) was evident. (TIF) [file pone.0115052.s003.tif]
