# Supplementary material for: Plant-Plant-Microbe Mechanisms Involved in Soil-Borne Disease Suppression on a Maize and Pepper Intercropping System
Source: PLoS One. 2014 Dec 31;9(12):e115052. doi: 10.1371/journal.pone.0115052 (PMC4281244; doi:10.1371/journal.pone.0115052)
Supplement: S1 Table — Disease control, yield and monetary value in monocultural and intercropping system. (DOCX) [file pone.0115052.s004.docx]

**Table S1 Disease control, yield and monetary value in monocultural and intercropping system**

| **Year** | **Crop** | **Variety** | **Yield±SE**  **(t/ha)** | **Crop value ±SE**  **(US$/ha)** | **LERs*** |
| --- | --- | --- | --- | --- | --- |
| 2009 | Maize | Haihe-2 | 6.10±0.05 | 1775.34±16.01 | — |
|  | Pepper | WJ-3 | 3.88±0.12 | 6275.47±187.52 | — |
|  | Intercropping | Haihe-2/WJ-3 | 6.64±0.06 | 7128.12±116.63 | 1.81 |
| 2010 | Maize | GY-135 | 7.36±0.10 | 2140.36±28.54 | — |
|  | Pepper | QB-1 | 3.62±0.12 | 5844.53±198.47 | — |
|  | Intercropping | GY-135/QB-1 | 6.80±0.11 | 6937.87±175.05 | 1.45 |
| 2011 | Maize | GY-135 | 7.18±0.13 | 2088.52±37.66 | — |
|  | Pepper | WJ-3 | 3.79±0.10 | 6119.25±488.14 | — |
|  | Intercropping | GY-135/WJ-3 | 6.70±0.12 | 7082.28±162.43 | 1.42 |

*Land equivalent ratios (LERs) were calculated as (yield ha^-1^ of maize in intercropping ÷ yield ha^-1^ of maize in monoculture) + (yield ha^-1^ of pepper in intercropping ÷ yield ha^-1^ of pepper in monoculture). Crop yield was determined by grain weight for maize and dry fruit weight for pepper. Crop values were based on market prices of 1616 US$ per ton for pepper, 291 US$ per ton for maize. Means and standard errors (SE) are shown.
