# Supplementary material for: Plant-Plant-Microbe Mechanisms Involved in Soil-Borne Disease Suppression on a Maize and Pepper Intercropping System
Source: PLoS One. 2014 Dec 31;9(12):e115052. doi: 10.1371/journal.pone.0115052 (PMC4281244; doi:10.1371/journal.pone.0115052)
Supplement: S2 Table — Compounds identified by GC/MS analysis in root exudates of maize variety Haihe-1 and Genyuan-135. (DOCX) [file pone.0115052.s005.docx]

**Table S2 Compounds identified by GC/MS analysis in root exudates of maize variety Haihe-1 and Genyuan-135**

| **CAS** | **Scientific name** | **Formula** | **Haihe-1** | | **Genyuan-135** | |
| --- | --- | --- | --- | --- | --- | --- |
|  |  |  | **Rention time**  **(min)** | **Confidence rate**  **（%）** | **Rention time (min)** | **Confidence rate**  **（%）** |
| 111-15-9 | 2-Ethoxyethyl acetate | C_6_H_12_O_3_ | 8.813 | 92.59 | 8.805 | 87.39 |
| 2040-76-8 | Carbamoyl chloride, phenyl- | C_7_H_6_CINO | — | —* | 10.230 | 92.30 |
| 62-53-3 | Aniline | C_6_H_7_N | — | — | 10.853 | 92.09 |
| 3173-53-3 | Cyclohexane, isocyanato- | C_7_H_11_NO | — | — | 11.431 | 84.99 |
| 78-40-0 | Triethyl phosphate | C_6_H_15_O_4_P | — | — | 15.574 | 81.19 |
| 95-16-9 | Benzothiazole | C_7_H_5_NS | 18.398 | 95.09 | 18.246 | 86.60 |
| 766-93-8 | Formamide, N-cyclohexyl- | C_7_H_13_NO | — | — | 19.270 | 80.78 |
| 2425-79-8 | Oxirane, 2’-[1,4-butanediylbis  (oxymethylene)]bis- | C_10_H_18_O_4_ | — | — | 27.768 | 83.69 |
| 2425-79-8 | Oxirane, 2,2’-[1,4-butanediylbis (oxymethylene)]bis- | C_10_H_18_O_4_ | 27.850 | 85.29 | — | — |
| 2254-94-6 | 3-methyl-2(3H)-  Benzothiazolethione, | C_8_H_7_NS_2_ | 27.916 | 87.3 | 27.991 | 81.37 |
| 615-22-5 | 2-(methylthio)-benzothiazole | C_8_H_7_NS_2_ | 28.171 | 90.59 | 28.121 | 90.59 |
| 28291-69-2 | 2-Benzothiazolamine, N-ethyl- | C_9_H_10_N_2_S | — | — | 29.061 | 68.98 |
| 934-34-9 | 2(3H)-Benzothiazolone | C_7_H_5_NOS | 29.779 | 87.39 | 29.816 | 88.39 |
| 489-84-9 | 1,4-Dimetyl-7-(1-methylethyl)-  azulene | C_15_H_18_ | 30.877 | 82.87 | 30.709 | 88.98 |
| 105794-58-9 | 1-Heptatriacotanol | C_37_H_76_O | — | — | 31.910 | 86.08 |
| 532-91-2 | 6-methoxy-2-benzoxazolinone | C_8_H_7_NO_3_ | 32.119 | 87.69 | 32.179 | 85.99 |
| 599-64-4 | Phenol, 4-(1-methyl-1- phenylethyl)- | C_15_H_16_O | — | — | 33.644 | 80.09 |
| 84-69-5 | 1,2-Benzenedicarboxylic acid, bis(2-methylpropyl) ester | C_16_H_22_O_4_ | 33.904 | 94.29 | — | — |
| 84-74-2 | Dibutyl phthalate | C_16_H_22_O_4_ | 35.787 | 96.096 | 35.652 | 93.30 |

*no compound was detected in this variety.
